# Supplementary material for: Meridianins Inhibit GSK3β In Vivo and Improve Behavioral Alterations Induced by Chronic Stress
Source: Mar Drugs. 2022 Oct 19;20(10):648. doi: 10.3390/md20100648 (PMC9605278; doi:10.3390/md20100648)
Supplement: Supplementary file 1 [file marinedrugs-20-00648-s001.zip › marinedrugs-1830081-supplementary.pdf]

Supplementary table

Table S1. Schematic organization of the chronic unpredictable mild stress protocol.

TIME	STRESSOR
Day 0	Isolation
Day 1	Light-dark cycle alterations (24 h)
Day 2	Forced Swim (5 min)
Day 3	Water deprivation (24 h)
Day 4	Exposition to rat sawdust (4 h)
Day 5	Home cage inclination (1 h)
Day 6	Water deprivation (24 h)
Day 7	Food deprivation (24 h)
Day 8	Light-dark cycle alterations (24 h)
Day 9	Forced Swim (5 min)
Day 10	Restrain (1 h)
Day 11	Exposition to rat sawdust (4 h)
Day 12	Home cage inclination (1 h)
Day 13	Food deprivation (24 h)
Day 14	Light-dark cycle alterations (24 h)
Day 15	Water deprivation (24 h)
Day 16	Forced Swim (5 min)
Day 17	Restrain (1 h)
Day 18	Exposition to rat sawdust (4 h)
Day 19	Light-dark cycle alterations (24 h)
Day 20	Home cage inclination (1 h)
Day 21	Water deprivation (24 h)
Day 22	Food deprivation (24 h)
Day 23	Restrain (1 h)
Day 24	Forced Swim (5 min)
Day 25	Exposition to rat sawdust (4 h)
Day 26	Restrain (1 h)
Day 27	Home cage inclination (1 h)
Day 28	Food deprivation (24 h)
